# EMOTIONS SURROUNDING THE 2018 MIDTERM ELECTIONS

**DOI:** 10.1093/geroni/igz038.1134

**Published:** 2019-11-08

**Authors:** Sydney Krueger, Kevin Ochsner

**Affiliations:** Columbia University, New York, New York, United States

## Abstract

Over the adult life-span there is a self-reported shift in daily life emotions towards feeling less negative and more positive. We hypothesized that variations in emotion regulation behavior over the life-span could explain why aging is associated with this “rosy glow”. We collected survey data from 400 adults on Prolific (18-90, M = 47, SD = 16) at three time points: once before and two times following the 2018 Midterm Elections. We collected political engagement ratings, baseline emotion ratings, emotion ratings following the election, and self-reported emotion regulation behavior (e.g., situation modification, situation selection, reappraisal, seeking social-support). In our analyses we treated age as a continuous variable predicting differences in emotion ratings and emotion regulation reports. Consistent with past research, age predicted a decrease in negative and an increase in positive emotions at baseline (before the election). Controlling for political affiliation and we found that age predicted a lower likelihood of using social support regulation and situation modification. We also found that age was inversely predictive of the use of multiple strategies, such that younger adults are more likely to rely on a larger array of regulatory strategies than older adults. These results suggest that age-related differences in self-reported emotions in daily life may be attributed to a reduction in regulation-strategy usage over the life-span, and perhaps a reduced need to regulate negative emotion.

